# Oleocanthal Quantification Using ^1^H NMR Spectroscopy and Polyphenols HPLC Analysis of Olive Oil from the Bianchera/Belica Cultivar

**DOI:** 10.3390/molecules26010242

**Published:** 2021-01-05

**Authors:** Martina Starec, Antonella Calabretti, Federico Berti, Cristina Forzato

**Affiliations:** Dipartimento di Scienze Chimiche e Farmaceutiche, Università degli Studi di Trieste, via L. Giorgieri 1, 34127 Trieste, Italy; martinastarec@gmail.com (M.S.); ANTONELLA.CALABRETTI@deams.units.it (A.C.); fberti@units.it (F.B.)

**Keywords:** olive oil, polyphenols, Folin-Ciocalteau, oleocanthal, *q*NMR, HPLC

## Abstract

The cultivar Bianchera is an autochthonous variety from the eastern part of northern Italy, but it is also cultivated in the Slovenian and Croatian peninsula of Istria where it is named Belica (Slovenia) and Bjelica (Croatia). The properties of oleocanthal, a natural anti-inflammatory ibuprofen-like compound found in commercial monocultivar extra virgin olive oils, were determined by means of both quantitative ^1^H NMR (*q*NMR) and HPLC analyses, where *q*NMR was identified as a rapid and reliable method for determining the oleocanthal content. The total phenolic content (TPC) was determined by means of the Folin–Ciocalteau method and the major phenols present in the olive oils were also quantified by means of HPLC analyses. All these analyses confirmed that the cultivar Bianchera was very rich in polyphenols and satisfied the health claim provided by the EU Commission Regulation on the polyphenols content of olive oils and their beneficial effects on human health.

## 1. Introduction

Extra virgin olive oil (EVOO) is one of the most valuable vegetable oils, part of the Mediterranean diet and is appreciated for its organoleptic properties, as well as for its nutritional value. Several benefits have been attributed to the consumption of olive oil, such as antioxidant properties, prevention in cardiovascular disease, chemoprevention, lower incidence of neurological disorders and neurodegeneration and protection towards inflammatory bowel diseases [1].

Most of these properties are due to the phenolic compounds present in extra virgin olive oil, such as hydroxytyrosol, oleuropein and oleocanthal, which are part of the 2% unsaponifiable fraction. This minor fraction contains several compounds of different classes, such as hydrocarbons (squalene, β-carotene), sterols (β-sitosterol, campesterol), fatty alcohols, triterpenes (uvaol, erythrodiol, maslinic acid, oleanolic acid), tocopherols, pigments, waxes and polyphenols (oleuropein, hydroxytyrosol, oleocanthal). Different from the other vegetable oils, EVOO is very rich in phenolic compounds since the olive fruit is a reservoir of them. Nevertheless, they are also present in the leaves of the *Olea europea* tree, as well as in pomace, the waste product of olive oil production. The presence of antioxidant compounds in waste products, such as leaves from pruning and pomace, means that there is a great interest in their recovery since natural extracts with antioxidant activity can be used as additives to enhance the quality and the duration of the product [2].

**Figure 1:**
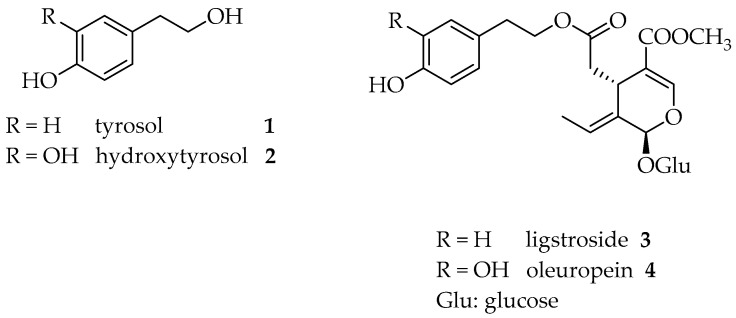
Chemical structures of phenols **1**–**4**.

The two phenolic alcohols, namely, tyrosol **1** and hydroxytyrosol **2**, as well as their derivatives (Figure 1), have received particular attention since they are considered natural antioxidants and, in 2011, their beneficial effects were recognised by the EFSA (European Food Safety Authority), which verified the cause and effect relationship between the consumption of polyphenols and the prevention of the lipoperoxidation of low density lipoproteins (LDL). In particular, a daily intake of at least 5 mg of polyphenols (tyrosol, hydroxytyrosol and their derivatives) provides these beneficial effects (EU Commission Regulation 432/2012).

The secoiridoid derivatives of tyrosol and hydroxytyrosol, namely, ligstroside **3** and oleuropein **4**, are only present in the plant family Oliaceae and they contain the elenolic acid skeleton, together with a glucose mojety (Figure 1) [3]. These compounds are responsible for some of the organoleptic properties of EVOO, such as bitterness and pungency.

**Figure 2:**
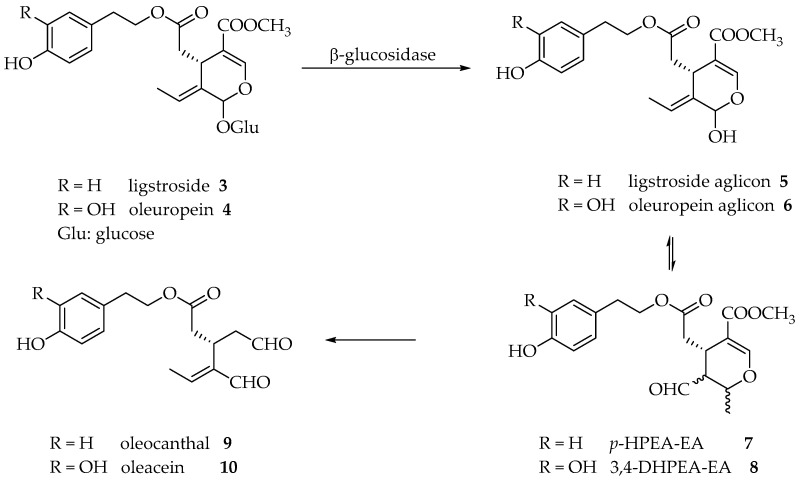
Transformation of ligstroside and oleuropein

**Figure 3:**
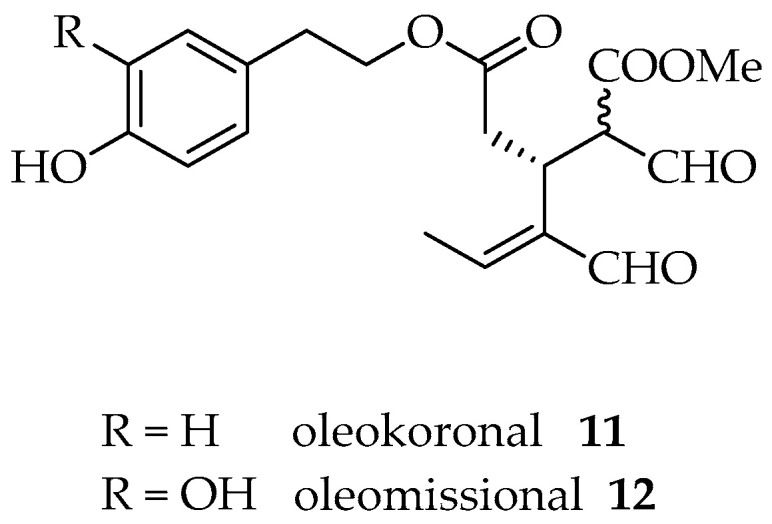
Chemical structures of oleokoronal and oleomissional.

Olive fruit is rich in oleuropein and ligstroside but during the extraction process, due to enzymatic or chemical reactions that can occur during the working process, they seem to be degraded first to ligstroside aglicon **5** and oleuropein aglicon **6** (by the action of β-glucosidases present in the skin and in the olive kernel) and second to the monoaldehydic forms *p*-HPEA-EA (*p*-hydroxyphenyl ethyl alcohol elenolic acid aldehyde) **7** and 3,4-DHPEA-EA (3,4-dihydroxyphenyl ethyl alcohol elenolic acid aldehyde) **8** as different diastereoisomers due to the ring opening and ring closure of the hemiacetal group (Figure 2). The open forms of the hemiacetals **5** and **6** were recently determined and named oleokoronal **11** and oleomissional **12**, respectively [4] (Figure 3). To obtain the two dialdehydic forms, namely, oleocanthal **9** and oleacein **10**, hydrolysis of the methyl ester and a decarboxylation reaction have to occur. These two steps could be reached either enzymatically or chemically due to the acidic aqueous conditions of the extraction. To our knowledge, no specific enzymes have been isolated till now [5].

The content of phenols in EVOO depends on several factors, such as the type of cultivar, climate, ripeness of the olives at harvesting, storage and the extraction process. The phenolic profile of cultivar Koroneiki from Greece was recently determined by Magiatis et al., who considered different factors, such as harvest time, malaxation temperature and malaxation time, where different trends were observed [6]. From the study of the malaxation process, the authors suggested a biosynthetic pathway for oleocanthal and oleacein through the formation of oleokoronal and oleomissional and a subsequent step of removal of the methyl group that is catalysed by a methylesterase. Furthermore, the same authors determined that these transformations are strongly dependent on the variety of olive oil.

Oleocanthal **9** and oleacein **10** have received significant attention in the last few years for their biological activity and chemical syntheses have been proposed [7,8,9,10]. These two molecules were first recognised by Montedoro in 1993 [11], and in particular, oleocanthal was successively extensively studied till, in 2005, Beauchamp confirmed it as a natural anti-inflammatory ibuprofen-like compound [12] due to its property of inhibiting the cyclooxygenases COX 1 and 2 activity. Several other biological activities have been recognised, for example, the attenuation of the markers of inflammation implicated in Alzheimer’s disease [13].

Several reviews are present in the literature regarding the analysis of polyphenols in olive oil [14,15], and apart from the Folin–Ciocalteau method, which is commonly used in several vegetable matrices to determine the overall content of polyphenols, many other techniques have been applied to determine the specific content of single polyphenols. HPLC and the best performing UHPLC are the most used techniques reported in the most recent literature [16,17,18,19], although spectroscopic methods have also been applied, such as near UV-Vis and NMR [20].

*Olea europaea* includes 35–40 species distributed over tropical and southern Africa, South Asia, eastern Australia, New Caledonia, and New Zealand [21]. The world’s olive germplasm contains more than 2629 different varieties, with many local varieties and ecotypes contributing to this richness; in Italy, about 800 cultivars are present [22,23]. The cultivar Bianchera is an autochthonous variety of Trieste, a city located in the eastern part of northern Italy, but it is also cultivated in the Slovenian and Croatian peninsula of Istria, where it is named Belica (Slovenia) and Bjelica (Croatia) [24,25]. Its characteristic is to be resistant to low temperatures and strong wind, typical of the city of Trieste, and since phenols are usually produced by the plant for protection against stress, in the present work, a complete characterisation of the phenols found in olive oil, olive leaves and pomace was performed. We focused our attention on commercial extra virgin olive oils to determine whether these olive oils could satisfy the EU health claim, although the phenolic content depends on several factors. Moreover, the analytical methods used as the extraction procedure, the instrument technique and the method of chemical expression have an important effect on the values obtained, as already observed by Pirisi et al. [26]. For these reasons, different extraction methods were applied and the total phenol content was determined using the Folin–Ciocalteau reagent. The oleocanthal quantification was made using the quantitative ^1^H NMR and the HPLC analysis for comparison purposes to verify whether these two techniques can furnish homogeneous results, as was already applied for the quantification of total hydroxytyrosol and tyrosol content in virgin olive oil [19]. Finally, we also measured the phenolic content in the leaves and pomace from Bianchera. This evaluation was carried out to evaluate the potential of such wastes as sources of phenols with the view of adding value to these products, which are currently not exploited in any way in the Trieste area. There is a growing interest in the use of aqueous extracts of olive leaves as food supplements, and on the other side, in the recovery of waste products, such as pomace.

## 2. Results and Discussion

### 2.1. Separation of the Secoiridoids Derivatives Using Column Chromatography

**Figure 4:**
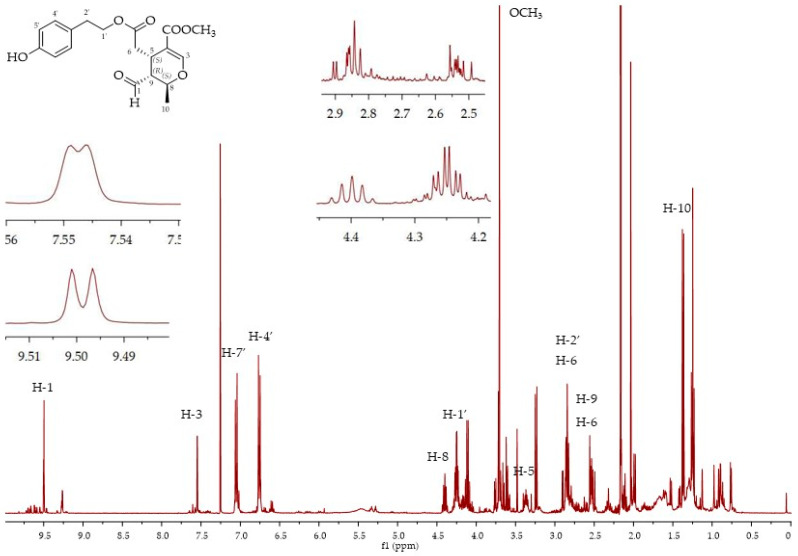
^1^H NMR spectra of (5*S*,8*R*,9*S*)-**7**.

**Figure 5:**
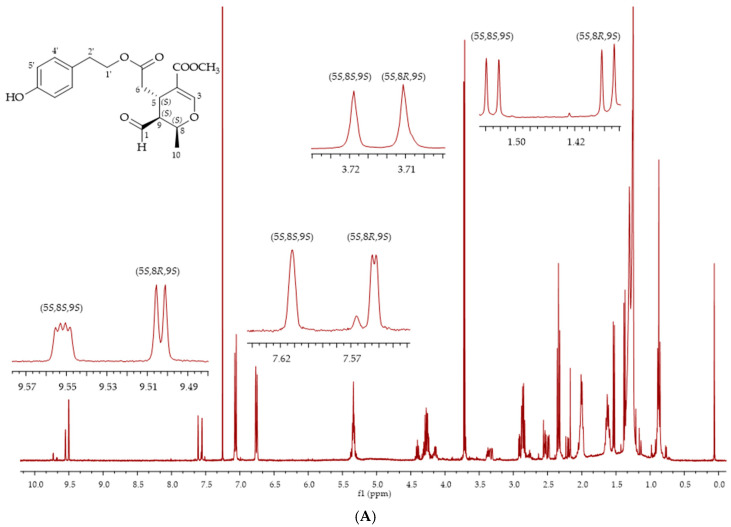

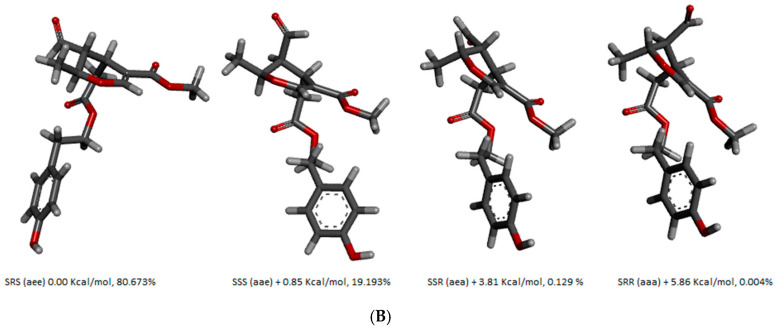
(**A**) ^1^H NMR spectra of the two diastereoisomers (5*S*,8*R*,9*S*)-**7** and (5*S*,8*S*,9*S*)-**7**. (**B**) Density functional theory (DFT)-optimised conformations of the four diastereoisomers of compound **7**, reporting the absolute configurations (axial/equatorial position of the side chains), relative energies and percentage of the population at room temperature.

In order to clearly identify compounds **7**–**10** using ^1^H NMR, we tentatively separated the secoiridoids using flash chromatography and we analysed the different fractions using both ^1^H NMR and LC-MS. Olive oil extract starting from 50 g of Bianchera olive oil was separated using column chromatography, as reported in the experimental part. It was possible to isolate seven different fractions, which were analysed and characterised using both ^1^H NMR and LC-MS. In the first fraction, the triacylglicerides components were found, while in the other six fractions, corresponding to different R_f_’s, it was possible to isolate the phenolic compounds. In fraction 2, using ^1^H NMR, it was possible to identify **7** as the only diastereoisomer (5*S*,8*R*,9*S*), although not as a pure compound (Figure 4). The assignment was made using ^1^H NMR signal comparison with the literature data for spectra registered in CDCl_3_ [27], although the same authors identified both diastereoisomers (5*S*,8*R*,9*S*)-**7** and (5*S*,8*S*,9*S*)-**7** via LC-SPE-NMR using CD_3_CN as the solvent for NMR analysis [28]. LC-MS confirmed this assignment since a peak at 32.3 min was observed with *m/z* 363 and 385, which corresponds to the [M + H]^+^ and [M + Na]^+^ of **7**. Using different elution conditions for the flash chromatography (starting from a less polar eluent), we also obtained the two diastereoisomers (5*S*,8*R*,9*S*)-**7** and (5*S*,8*S*,9*S*)-**7**, indicating that an equilibrium was present between ligstroside aglicon **5** and the aldehydic form of ligstroside **7**, leading to yields of two of the four diasteroisomers that can be formed (Figure 5A). In these conditions, the longer time of contact with the silica gel probably led to the formation of both diastereoisomers. We have verified through carrying out a density functional theory (DFT) calculation (b3lyp/6.31 G(d,p)/PCM polarizable continuum model of solvent) that the 5*S*,8*R*,9*S*-**7** and 5*S*,8*S*,9*S*-**7** are actually the most stable among the four possible diastereoisomers (Figure 5B). The configurations of the 5*S*,8*R*,9*S*-**7** isomer allows for keeping all the side chains in a pseudo-equatorial orientation, while in the 5*S*,8*S*,9*S*-**7** isomer, the aldehydic group at position 9 becomes pseudo-axial.

**Figure 6:**
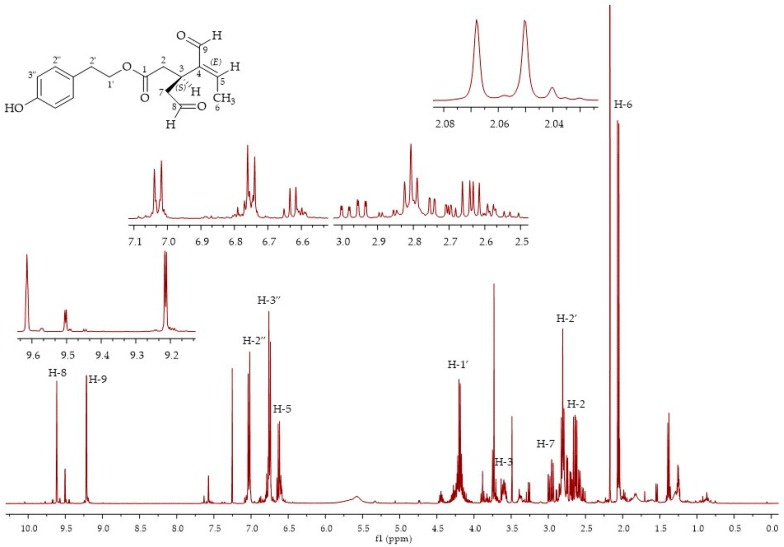
^1^H NMR spectra of fraction 3, oleocanthal (3*S*,4*E*)-**9**.

**Figure 7:**
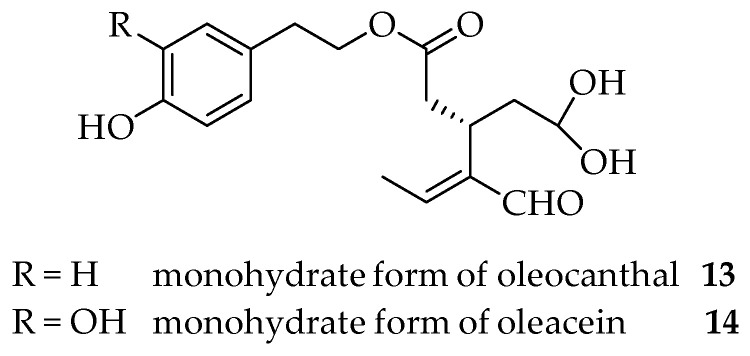
Monohydrate forms of oleocanthal **13** and oleacein **14**.

In fractions 3 and 4, it was possible to isolate oleocanthal **9**, although in an admixture with compound **7** in fraction 3, and compound **8** in fraction 4. The oleocanthal signals were in accordance with the literature data for the diastereoisomer (3*S*,4*E*), with the characteristic peak for the methyl group at the double bond at 2.08 ppm and the aldehydic protons at 9.63 and 9.22 ppm [29] (Figure 6). LC-MS analyses of the third fraction showed a broad peak at 28.6 min for oleocanthal **9** (*m*/*z* 327, [M + Na]^+^) due to the presence of the monohydrate form of oleocanthal **13** (*m*/*z* 345, [M + Na]^+^) (Figure 7) and a peak at 30.6 min for compound **8** (*m/z* 379, [M + H]^+^; *m*/*z* 401, [M + Na]^+^). The presence of the monohydrate forms of the secoiridoids has already been presented in the literature since the hydrated dialdehydic form of oleuropein was detected as a stable compound in water [27].

**Figure 8:**
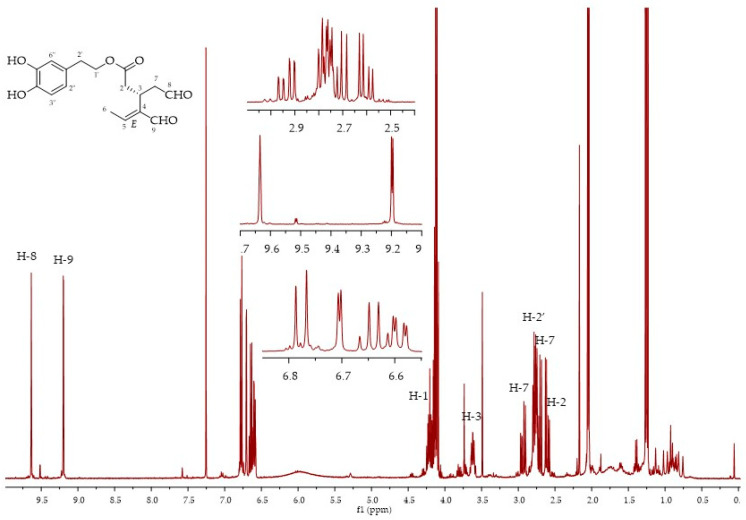
^1^H NMR spectra of fraction 7, oleacein (3*S*,4*E*)-**10**.

Fraction 5 was a mixture of compounds **8**, **9** and **10**. Fraction 6 was a mixture of compounds **8** and **10**, while fraction 7 contained mainly oleacin **10,** although not as a pure compound, as confirmed via ^1^H NMR and LC-MS analyses (Figure 8). The two characteristic peaks of the aldehydic protons of **10** at 9.57 (bs) and 9.14 (d) were present in the ^1^H NMR and a broad peak at 23–25 min was present in the LC-MS chromatogram. Furthermore, in this case, the mass spectrum showed that oleacein (*m/z* 343, [M + Na]^+^) was present in the admixture with its monohydrate form **14** (*m/z* 361, [M + Na]^+^).

**Figure 9:**
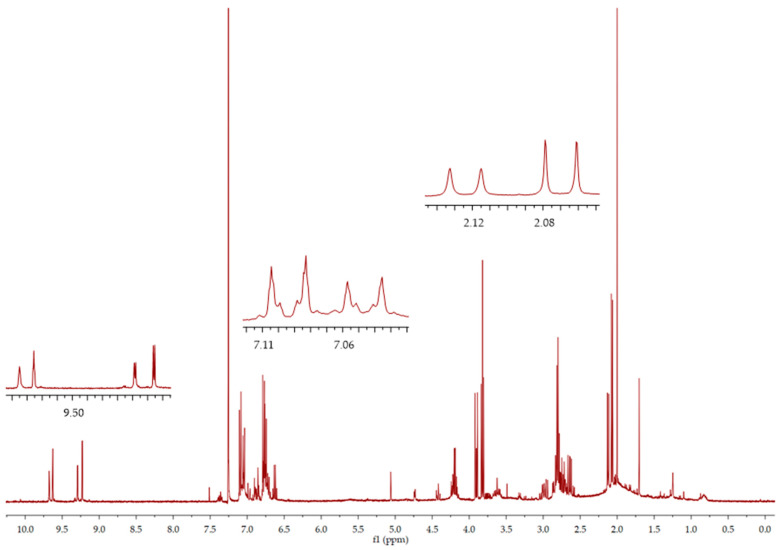
^1^H NMR spectra of oleocanthal **9**.

**Figure 10:**
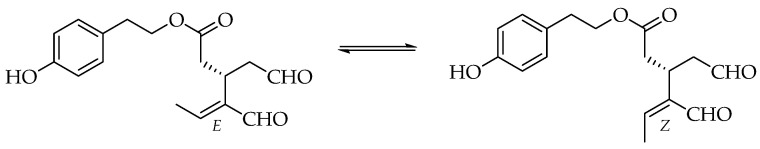
Oleocanthal interconversion.

Since it was not possible to isolate pure compounds using column chromatography, we decided to further separate the column fractions via semi-preparative HPLC using a C18 column and a mixture of water-acetonitrile as the eluent. Four different fractions were collected, corresponding to the following retention times: 18.4 min (oleacein **10**), 21 min (oleocanthal **9**), 22.7 min (*p*-HPEA-EA **7**) and 25 min (3,4-DHPEA-EA **8**). After lyophilisation of the samples, ^1^H NMR spectra were recorded. The fraction corresponding to oleocanthal **9** showed the presence of two compounds in an admixture, with the two characteristic aldehydic protons at 9.23 (d) and 9.63 (s) for oleocanthal, and at 9.17 (d) and 9.58 (s) for the other unknown compound. (Figure 9) Two doublets were also present at 2.07 ppm and 2.01 ppm, corresponding to the methyl group of the double bond. As already observed in the literature, the two compounds were the isomers *E* and *Z* of oleocanthal [28]. During the HPLC separation, an interconversion probably occurred from isomer *E* to isomer *Z* through a Michael addition/elimination process, as proposed in the literature [29] (Figure 10).

Fractions corresponding to oleacein **10** and *p*-HPEA-EA **7** were not pure compounds but a mixture of two or more compounds that were probably formed during the HPLC separation.

### 2.2. Quantification of Oleocanthal and Oleacein Using qNMR

Different extraction methods are reported in the literature for the extraction of phenols from olive oil [30,31,32,33,34] and an 80:20 mixture of methanol-water is generally used, although other solvents can be exploited, such as acetonitrile, ethanol and other hydroalcoholic mixtures [35]. Besides these classical extractions, other extractions protocols have been used, such as microwave- and ultrasound-assisted ones [33]. Moreover, since olive oil is principally made of lipids, it is possible to add a defatting solvent, such as hexane or cyclohexane, to facilitate the extraction of polyphenols. Different results are obtained in the literature depending on the extraction mode and the kind of analysis; therefore, we decided to evaluate both extraction methods as different analysis methods of the olive oil of cultivar Bianchera in order to verify whether this kind of cultivar satisfies the health claim condition.

To establish the different extraction method capacities, we quantified oleocanthal **9** and oleacein **10** contents using quantitative ^1^H NMR since the aldehydic protons of these two compounds are in the range of 9–10 ppm and can be well distinguished. As the internal standard, we used 4-nitrobenzaldehyde, which shows the aldehydic proton resonance at 10.15 ppm (Table 1), and as the first extraction procedure, we chose the Karkoula method, which is identified as method A in Table 2 [36].

**Figure 11:**
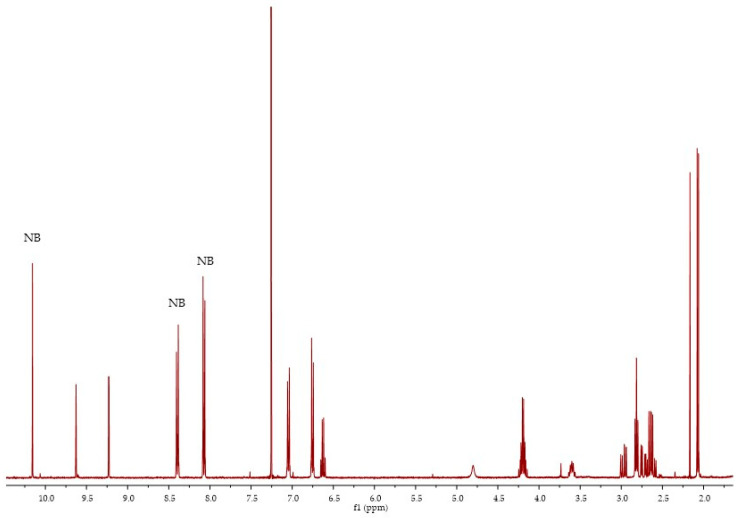
^1^H NMR spectra of oleocanthal **9** in a 1:1 admixture with 4-nitrobenzaldehyde (NB).

To quantify compounds **9** and **10**, a calibration curve was built using commercial oleocanthal as a pure standard compound. In Figure 11, the ^1^H NMR spectra in CDCl_3_ of oleocanthal (*E* isomer) and 4-nitrobenzaldehyde (NB) in an admixture are reported. Since **9** and **10** differ only by one OH group, the same calibration curve was also used for the quantification of **10**. In fact, in the literature, calibration curves of **9** and **10** using *q*NMR are very similar [36]. The quantification of compounds **7** and **8** was also done using the same calibration curve since the structure is very similar and standard compounds are not available.

In Table 1, the chemical shifts (c.s.) of the aldehydic protons of compounds **7**–**10** are summarised.

Oleocanthal **9** and oleacein **10** have two different aldehydic protons, which were present in the ^1^H NMR spectra as doublets, but the ones at 9.23 and 9.21 ppm were partially overlapped by other signals that are usually not observed in the literature. This overlapping was present in the literature only in very few cases but the cultivars were different (less than 3%) [30].

Different extraction conditions were evaluated to establish which was the better one in order to obtain the higher values of compounds **7**–**10** that contribute to a satisfactory health claim condition.

Usually, the extraction solvent is a mixture of methanol and water, which is also used in the official method of the International Olive Council (IOC) [37], where the analyses are performed by HPLC-UV or HPLC-MS, but the formation of several artefacts was observed, which made the quantification very difficult. It is well known that oleocanthal and oleacein react with water and methanol, which are often used as the mobile phase in the HPLC analysis, leading to the corresponding acetals and hemiacetals. All these artifacts give complex chromatograms, making quantification difficult. This is the reason why Karkoula used acetonitrile as the extraction solvent. This aspect was reconsidered in 2017 by Priego-Capote [38], whose studies established that the use of methanol-water solutions for phenol extraction was not influential on the formation of these artefacts.

Together with the Karkoula method, we also considered extractions with methanol-water mixtures and the use or not of a defatting solvent. The results are summarised in Table 2, where the concentrations of compounds **7**–**10** are reported in milligrams per kilogram of olive oil (mg/kg) for comparison with the literature data. Method A corresponds to the Karkoula method. The standard deviation was around 10% for compounds **8**–**10**, while for oleacein **7**, it was around 20%.

If we compare the results obtained using method A with the results obtained by Karkoula, although for different olive oils, the value of 525 mg/kg found as the sum of the four secoiridoids was in the range found by Karkoula, whose values ranged from not detectable to 1534 mg/kg [30].

The difference between method A and B was only in the volume of solvent used for the extraction since in method B, the volume was halved but the efficiency of the extraction was the same. This result is encouraging since only a consistently minor volume of an organic solvent needs to be used to obtain the same results. Furthermore, the best results were obtained with a hydroalcoholic mixture as the extraction solvent instead of acetonitrile, which enhanced the extraction efficiency but it strongly depended on the MeOH/H_2_O ratio used, as an 80/20 ratio dramatically enhanced the secoiridoids extraction (method C), confirming the results obtained by Tsimidou et al. [35] (Table 2). Method E was also a good extraction method but it seemed that the defatting solvent played an important role in the efficiency of the secoiridoid extraction since a total of 2335 mg/kg was extracted compared to 3193 mg/kg with method C.

**Figure 12:**
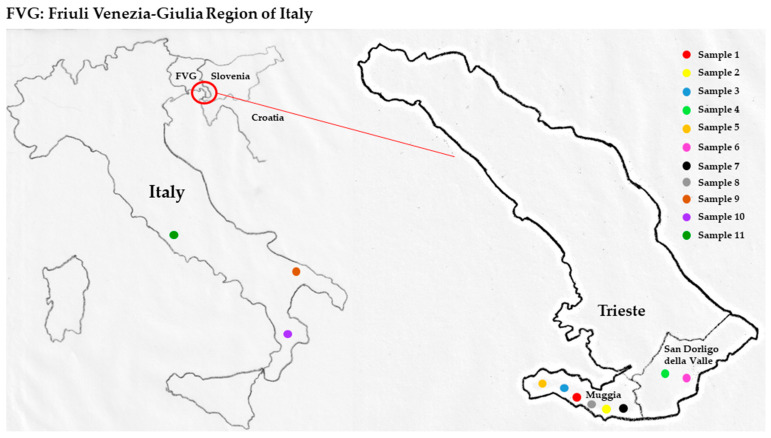
Olive oil samples’ geographical distribution.

We considered 12 samples of olive oils that were analysed to determine the total phenolic content (Section 2.4), while for *q*NMR, we only analysed samples 2 and 6 of the Bianchera cultivar with a different geographical origin, and sample 12 (commercial olive oil from EU of unknown varietal composition) (Table 3 and Figure 12). Method C was used for the extraction and analyses of the three different olive oil samples are reported in Table 4.

Samples 2 and 6 of the cultivar Bianchera had double the amount of secoiridoids relative to sample 12 of unknown varietal composition. (Table 4) Moreover, olive oils of cultivar Bianchera had an amount of oleocanthal of about 50%, while sample 12 had only 35%. The higher percentage of oleocanthal in Bianchera olive oil gives a higher value to this olive oil due to the anti-inflammatory properties of this secoiridoid.

**Figure 13:**
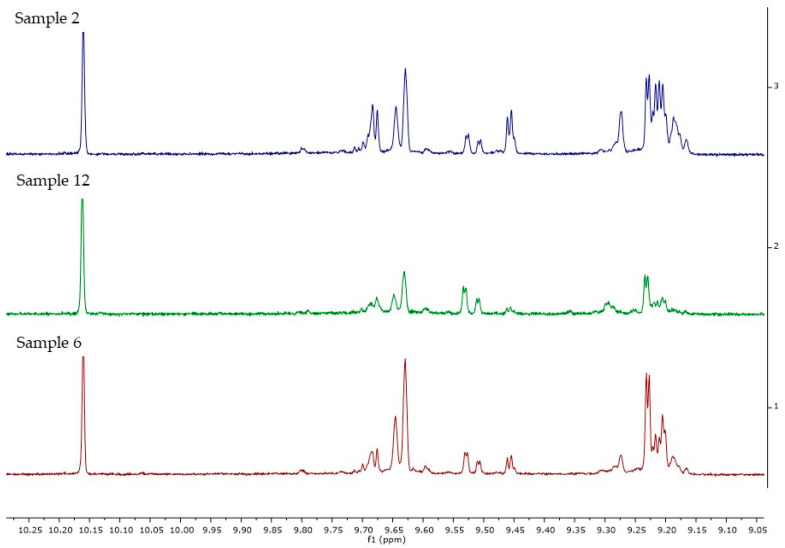
Enlargement of the aldehydic zone of ^1^H NMR for samples 2, 6 and 12.

In Figure 13, the ^1^H NMR spectra for the three samples (2, 6 and 12) are reported, where it is evident that the amount of compounds present in the 9.00–9.80 ppm zone of the spectrum, related to the aldehydic protons, are higher for samples 2 and 6. In these two samples from cultivar Bianchera, it can also be noticed that there are more complicated signals around 9.2 ppm, which are not already completely assigned.

To understand whether quantification via ^1^H NMR of oleocanthal is a good method, the same olive oil samples were analysed via HPLC using a calibration curve of commercial oleocanthal, where the results are reported in Table 4. As it can be seen from Table 4, the results are in good agreement, meaning that ^1^H NMR can be considered a good method for the quantification of oleocanthal. Although it requires an expensive instrument, this procedure is quick and no large quantities of solvents are needed to perform the analysis.

### 2.3. Quantitative HPLC Analyses

To verify whether the olive oil extracts from cultivar Bianchera satisfy the Health Claim condition, quantitative HPLC analyses were performed on olive oil samples 2, 6 and 12 using a calibration curve of tyrosol and expressing the results in milligrams of tyrosol equivalent per mL (mg(TE)/mL).

In Table 5, the results expressed as milligrams of tyrosol equivalent per 20 g of olive oil (mg(TE)/20 g olive oil) are reported in order to directly compare the values obtained with the health claim indications.

From the results obtained, both olive oils from the Bianchera cultivar (sample 2 and 6) satisfied the health claim condition since more than 5 mg of hydroxytyrosol and its derivatives were present (6.52 mg(TE)/20 g for sample 2 and 6.93 mg(TE)/20 g for sample 6), while sample 12 of unknown varietal composition has lower content. It is important to note that samples 2 and 6 of Bianchera cultivar had a higher content of secoiridoids, while sample 12 was richer in tyrosol and hydroxytyrosol, probably due to a hydrolysis reaction that occurred to realise the two simple phenols from the secoiridoids oleuropein and ligstroside.

### 2.4. Determination of the Total Phenolic Content

The quantification of the polyphenols content in EVOO is becoming important in the definition of the EVOO quality and allows for labelling according to EU standards. The determination of the total phenolic content (TPC) was made by means of the Folin–Ciocalteau method, which was developed in 1972 for the determination of the aminoacids tyrosin and tryptophan and subsequently used for the quantification of the total phenols in different matrices [39]. The Folin–Ciocalteau method was applied in the analysis of 12 olive oils, comprising 6 olive oils of monocultivar Bianchera and 6 olive oil of other origins, as reported in Table 3, to demonstrate the higher content of phenols attributed to the Bianchera cultivar (Figure 12).

The quantitative determination was made by using a calibration curve with a standard solution of gallic acid (Supplementary Material Figure S4).

The samples obtained using the extraction procedure reported in Section 3.9 were diluted 1:10 prior to the analysis. The results are reported in Table 6 and are expressed in milligrams of gallic acid equivalent per kilogram (mg(GAE)/kg) and in milligrams of gallic acid equivalent per 20 g of olive oil (mg(GAE)/20 g olive oil) to see whether the value can satisfy the health claim condition, although the health claim considers only the content of hydroxytyrosol and its derivatives.

The total phenolic content cannot be used in order to label the EVOO according to the health claim, which may be used only for olive oil containing at least 5 mg of hydroxytyrosol and its derivatives (e.g., oleuropein complex and tyrosol) per 20 g of olive oil since in the total phenols content, phenolic acids, cinnamic acids, flavonoids and lignans are included. The only official method that can be used to satisfy the health claim is the IOC method [37] but other methodologies have been proposed since quantification via HPLC is difficult because of many compounds overlapping. Moreover, it is not clear which derivatives must be considered. In any case, the total phenolic content can be in the first instance a good parameter to verify whether an EVOO could satisfy the health claim.

As it can be seen from Table 6, all olive oils of the Bianchera cultivar (samples 1 to 6) had a TPC higher than 5 mg/20 g, while for the other cultivars, only two had a good value (samples 7 and 9). This was in accordance with what is already presented in the literature [40,41,42].

Furthermore, the pomace and olive leaves extractions were analysed and their total phenolic contents were determined. For the extraction of pomace, fresh pomace was used, with a water content of 67–68%. Three different solvent mixtures were used: MeOH/H_2_O 80/20; EtOH/H_2_O 80/20 and EtOH/H_2_O 30/70. The averages of the triplicate analyses were 3010 ± 120 of TPC mg(GAE)/kg for the pomace extraction with MeOH/H_2_O 80/20, 3240 ± 40 of TPC mg(GAE)/kg for the pomace extraction with EtOH/H_2_O 80/20 and 3510 ± 270 of TPC mg(GAE)/kg for the pomace extraction with EtOH/H_2_O 30/70. All extractions gave similar results with a TPC ranging from 3010 to 3510 mg(GAE)/kg, where the higher value was obtained with a higher content of water. Furthermore, the values obtained are not directly comparable with literature results since the extraction method was different and usually TPC values are expressed as milligrams of gallic acid equivalent per 100 g of oil (mg(GAE)/100 g oil) for pomace [43,44].

Moreover, for the extraction of olive leaves, three different solvent mixtures were used: MeOH/H_2_O 80/20; EtOH/H_2_O 70/30 and H_2_O 100%. The averages of triplicate analyses for the olive leaves extraction were 42.8 ± 2 of TPC mg(GAE)/g with MeOH/H_2_O 80/20, 37.3 ± 0.8 of TPC mg(GAE)/g with EtOH/H_2_O 70/30 and 18.7 ± 0.7 of TPC mg(GAE)/g with only water. For the olive leaf extracts, the value of 42.8 ± 2 mg(GAE)/g is in accordance with the values reported in the literature (from 42.35 ± 0.002 mg(GAE)/g to 190.65 ± 0.03 mg(GAE)/g), although we reported the values in mg(GAE)/g (leaves), while in the literature, they are reported in mg(GAE)/g (dry extract) [45]. In any case, it is evident that the leaves of the Bianchera cultivar are rich in polyphenols and can be considered as a good source of these compounds. Moreover, from our previous work [46], which was aimed at sensing phenols in aqueous olive leaves extracts by means of fluorescent imprinted nanoparticles, we verified via HPLC that the main polyphenol present in aqueous olive leaves extracts was oleuropein.

### 2.5. Antioxidant Power (Trolox Equivalent Antioxidant Capacity (TEAC)/ABTS Assay)

We also determined the antioxidant power of the olive oil extracts by means of the ABTS test and TEAC, which compare the total antioxidant capacity of the sample with the one of Trolox, a synthetic analogue of vitamin E, using the method reported by Re et al. [47]. The calculation of TEAC values is a little bit confusing in the literature since values are reported using different units and it is not clear how it is calculated. For the sake of clarity, herein, we report the TEAC as the ratio between the gradient of the plot of the percentage inhibition of the absorbance vs. the concentration plot for the sample and the gradient of the plot of Trolox using the same units. Therefore, the TEAC value is a dimensionless value. The results obtained for olive oil samples 2, 6 and 12, the pomace extracts and the olive leaves extracts are reported in Table 7.

As can be seen from Table 7, the TEAC/ABTS values of the three olive samples indicated that the olive oil from the Bianchera cultivar (sample 2 and 6) had a higher antioxidant activity, as well as a higher content of phenolic compounds. In the case of pomace, the values of TPC and TEAC/ABTS indicated that although both the use of the MeOH/H_2_O 80/20 and EtOH/H_2_O 30/70 mixtures showed the best extraction of polyphenols, the higher value of TEAC was obtained for the extraction with EtOH/H_2_O 80/20, suggesting that this is the best mixture to extract polyphenols with the higher characteristics as antioxidants.

In contrast, for leaves, the extraction with MeOH/H_2_O 80/20 gave a higher level of phenols content, as well as the higher antioxidant activity.

Extracts from pomace and olive leaves in the three different extraction conditions were analysed via HPLC in order to quantify the major compounds present. In the HPLC chromatograms of the pomace extraction, mainly tyrosol and hydroxytyrosol were found, which were quantified using a calibration curve of tyrosol. Tyrosol was in the range of 0.120–0.135 mg/mL while hydroxytyrosol was in the range of 0.025–0.030 mg/mL, depending on the solvent used for extraction.

In olive leaves extracts, the main compound was oleuropein with lower amounts of tyrosol and hydroxytyrosol. Extraction with 80/20 MeOH/H_2_O gave 38.49 mg/g (leaves) of oleuropein, while extraction with water gave only 0.07 mg/g (leaves). Tyrosol and hydroxytyrosol were present in all solvent extractions, with quantities ranging from 0.15 to 0.69 mg/g (leaves).

Since the TEAC values for pomace were higher relative to the values obtained for leaves, other phenols contributed to the antioxidant activity apart from oleuropein, tyrosol and hydroxytyrosol. Comparison with literature data for olive oils [48], pomace [49] and olive leaves [50] is not simple since each one reports the TEAC values with different units.

## 3. Materials and Methods

### 3.1. Chemicals and Materials

Hydroxytyrosol and oleuropein were purchased from Carbosynth (Compton, U.K.). All the other reagents were purchased from Sigma-Aldrich (Milano, Italy). All solvents were of HPLC grade. The silica gel was 60 Å, 230–400 mesh, while the TLC was silica gel 60 F254 on PET.

Eight out of the 12 olive oils used were furnished by local farmers of the Region Fiuli-Venezia Giulia (Italy), while 3 olive oils were commercial olive oils from different parts of Italy and one commercial olive oil from the EU. The olive oils used are listed in Table 3.

Pomace and olive leaves were furnished by a local farmer in Trieste and were from the Bianchera cultivar.

### 3.2. Instrumentation

NMR spectra were recorded on Varian 400 or 500 MHz spectrometers (Palo Alto, CA, USA). HPLC analyses were run on an Agilent 6120-Infinity liquid chromatograph (Santa Clara, CA, USA) or an Agilent 6210 Infinity II chromatograph equipped with a Kinetex C18 150 × 2.1 mm, 5 μm, 100 Å (Phenomenex, Torrance, CA, USA) column with a column guard and an autosampler using a UV detector at 280 nm. The flow was set to 0.25 mL/min. Spectrophotometric measurements were run on a UV-Vis Cary-60 (Agilent) (Santa Clara, CA, USA) at 765 nm for the phenolic content and at 734 nm for the ABTS test.

Lyophilisations were performed on a Scanvac CoolSafe lyophiliser at −55 °C and 0.9 mbar.

### 3.3. Olive Oil Extraction

To optimise the extraction method, several methods were tested, and extra virgin olive oil from a local farmer of the city of Trieste (Italy) was used (80% Bianchera, 20% Pendolino + Leccino).

Method A: This method was from the literature [36]. In a 50 mL centrifuge tube, 5 g of olive oil was diluted with 20 mL of hexane and 20 mL of acetonitrile. The mixture was stirred with an Analog Vortex mixer (VWR International, Milano, Italy) for 30 s and successively centrifuged for 5 min at 4000 rpm. After the separation of the two phases, the phase in acetonitrile was evaporated with the rotary evaporator.

Method B: In a 50 mL centrifuge tube, 5 g of olive oil was diluted with 10 mL of hexane and 10 mL of acetonitrile. The mixture was stirred with a Vortex system for 30 s and the two phases were separated. The phase in acetonitrile was evaporated using a rotary evaporator (DLAB RE 100-S, DLAB Scientific Europe S.A.S, Schiltigheim, France).

Method C: In a 50 mL centrifuge tube, 5 g of olive oil was diluted with 10 mL of hexane and 10 mL of a methanol-water 80:20 mixture. The mixture was stirred with a Vortex system for 30 s and the two phases were separated. The phase in methanol-water was evaporated using a rotary evaporator.

Method D: In a 50 mL centrifuge tube, 5 g of olive oil was diluted with 10 mL of hexane and 10 mL of a methanol-water 50:50 mixture. The mixture was stirred with a Vortex system for 30 s and the two phases were separated. The phase in methanol-water was evaporated using a rotary evaporator.

Method E: In a 50 mL centrifuge tube, 5 g of olive oil was diluted with 10 mL of petroleum ether and 10 mL of acetonitrile. The mixture was stirred with a Vortex system for 30 s and the two phases were separated. The phase in acetonitrile was evaporated using a rotary evaporator.

### 3.4. Pomace Extraction

In a 50 mL centrifuge tube, 5 g of dry pomace was diluted with 10 mL of a MeOH/H_2_O 80/20 mixture. The mixture was stirred with a Vortex system for 30 s and successively sonicated for 15 min. After vacuum filtration, the hydroalcoholic phase was extracted with 10 mL of petroleum ether. The hydroalcoholic phase was concentrated using a rotary evaporator and the volume was adjusted to 10 mL.

The same procedure was used with EtOH/H_2_O 80/20 and EtOH/H_2_O 30/70 mixtures as the extraction solvents.

### 3.5. Olive Leaves Extraction

Olive leaves were air-dried for 24 h, immersed in liquid nitrogen and crushed in a mortar until they were smaller than 1 cm^2^.

A mixture of 1 g of olive leaves and 10 mL of a MeOH/H_2_O 80/20 mixture was stirred in the dark for 24 h at room temperature. After vacuum filtration on a paper filter, the hydroalcoholic mixture was centrifuged at 4000 rpm for 25 min and the supernatant was taken for the analyses.

The same procedure was used with EtOH/H_2_O 70/30 and H_2_O 100% mixtures as the extraction solvents.

### 3.6. Separation by Column Chromatography

A 50 g sample of Bianchera olive oil was extracted using method C to obtain 70 mg of olive oil extract, which was purified using column chromatography. A 1.0 cm diameter column with silica gel as the stationary phase was used (20 cm length of silica gel). A gradient was used, starting from petroleum ether/ethyl acetate 60/40 and adding 5% of ethyl acetate for every 50 mL of the eluent. TLC analyses were performed with a mixture of petroleum ether/ethyl acetate 40/60. To recognize the phenols, TLC plates were sprayed with an aqueous solution of Na_2_CO_3_, dried and subsequently sprayed with a diluted solution of the Folin–Ciocalteau reagent.

### 3.7. Quantification Using ^1^H NMR (qNMR)

To the extract of olive oil, 1 mL of a solution of internal standard (IS) in acetonitrile with a concentration of 5.955 × 10^−3^ M (corresponding to 5.955 μmol of 4-nitrobenzaldehyde) was added and the solvent was evaporated. The mixture of olive oil extract with the internal standard was dissolved in 800 μL of CDCl_3_ and 700 μL of the obtained solution was transferred into an NMR tube.

For the extracts obtained from the pomace and the olive leaves, which were not completely soluble in CDCl_3_, it was necessary to dissolve the extracts in chloroform and filter the solid. The organic phase was evaporated and the obtained residue was dissolved in CDCl_3_.

To quantify compounds **9** and **10**, a calibration curve was built to report the integration signal ratio oleocanthal/IS versus oleocanthal(mg)/IS(mg) (*y* = 0.2715*x* + 0.015) using commercial oleocanthal as a pure standard compound. The calibration curve for oleocanthal showed good response linearity with a correlation coefficient (*R*^2^) of 0.999. Oleocanthal(mg)/IS(mg) was in the range of 0.5–3.5.

### 3.8. HPLC Analyses

Qualitative HPLC analyses were performed on an LC-MS Agilent 6210 Infinity chromatograph that was coupled with a mass spectrometer equipped with a Kinetex C18 150 × 2.1 mm, 5 μm, 100 Å column (Phenomenex) with a column guard and an autosampler using a UV detector at 220 nm, 254 nm and 280 nm. The flow was set to 0.25 mL/min. The injection volume was 5 μL.

The eluent was solvent A: H_2_O + 0.05% TFA and solvent B: CH_3_CN + 0.05% TFA using the gradient found in Table 8.

Semi-preparative HPLC analyses were performed on an Agilent 6210 Infinity I chromatograph equipped with a Kinetex C18 250 × 10.0 mm, 5 μm, 100 Å column (Phenomenex). The flow was set to 3.0 mL/min. The injection volume was 500 μL. The eluent was solvent A: H_2_O + 0.05% TFA and solvent B: CH_3_CN + 0.05% TFA using the gradient found in Table 9.

Analyses of the olive oil, pomace and olive leaves extracts were performed on samples prepared in water at the concentrations of 0.5 g/mL for olive oil, 0.4 g/mL for pomace and 0.1 g/mL for olive leaves. The solutions were filtered on 0.45 μm PTFE filters prior to injection.

Quantitative HPLC analyses were performed on an Agilent 6210 Infinity II chromatograph equipped with a Kinetex C18 250 × 10.0 mm, 5 μm, 100 Å column (Phenomenex) using the same method as for the qualitative HPLC analyses. Detection was made using a UV detector at 220 nm. To quantify compounds **1, 2, 7, 8** and **10**, a calibration curve for tyrosol was built (*y* = 41729*x* + 15.349). The calibration curve for tyrosol showed good response linearity with a correlation coefficient (*R*^2^) of 0.999.

To quantify compound **9** (oleocanthal), a calibration curve for oleocanthal was built (*y* = 5797.5*x* + 69.541). The calibration curve for oleocanthal showed good response linearity with a correlation coefficient (*R*^2^) of 0.997.

### 3.9. Total Phenolic Content

To determine the total phenolic content for olive oils, the extraction method was performed as follows: In a 50 mL centrifuge tube, 5 g of olive oil was diluted with 10 mL of hexane and 10 mL of an 80:20 methanol-water mixture. The mixture was sonicated for 15 min and the two phases were separated. The phase in methanol-water was evaporated using a rotary evaporator and the residue was diluted in 10 mL of water.

Mother solutions of olive oil, pomace and olive leaves extracts in water at the concentrations of 0.5 g/mL for olive oil, 0.4 g/mL for pomace and 0.1 g/mL for olive leaves were diluted at concentrations of around 50 mg/mL for olive oil extracts, 40 mg/mL for pomace and 20 mg/mL for olive leaves.

The quantitative determination was made by using a calibration curve with a standard solution of gallic acid. The calibration curve for gallic acid (*y* = 7.8772*x* + 0.0337) showed good response linearity with a correlation coefficient (*R*^2^) of 0.996.

In a 10 mL volumetric flask, 1 mL of the solution to be analysed was added together with 0.5 mL of the Folin–Ciocalteau reagent (2N), and after 3 min, 1 mL of a 10% Na_2_CO_3_ solution in water was added and the volume was adjusted to 10 mL. After 1 h in the dark, the solution was transferred in a cuvette for the UV measurement.

### 3.10. TEAC/ABTS Assay

A total of 50 μL of a K_2_S_2_O_8_ solution (140 mM) was added to 950 μL of an ABTS solution (7 mM) and the mixture was kept in the dark for 16 h. The obtained solution of ABTS^+·^ was subsequently diluted with ethanol to have an absorbance at 734 nm of 0.70 ± 0.02.

To evaluate the steady state required to reach the complete radical-scavenging reaction, the following samples were used:

For olive oil, extract sample 2 was used with a concentration of 0.0203 mg GAE/mL;

For pomace, an extract sample obtained via ethanolic extraction was used with a concentration of 0.0351 mg GAE/mL;

For olive leaves, an extract sample obtained via aqueous extraction was used with a concentration of 0.375 mg GAE/mL.

A 50 μL sample of the olive oil extract (0.0203 mg GAE/mL) was added to 950 μL of the ABTS^+·^ solution and the absorbance (A) was measured at regular intervals until the absorbance was constant. The inhibition percentage (I%) was calculated according to the following formula:

(1) $$I\%=\frac{A\left(\mathrm{ABTS}\right)-A\left(\mathrm{sample}\right)}{A\left(\mathrm{ABTS}\right)}\times 100.$$

For the olive oil extract, the steady state was reached after 40 min.

For the pomace extract, the steady state was reached after 80 min.

For the olive leaves extract, the steady state was reached after 60 min.

A 50 μL sample of each solution was mixed with 950 μL of ABTS and the mixture was kept in the dark for the time necessary to reach the steady state. The absorbance was measured and calibration curves were built after 40 min for the olive oils, 80 min for the pomace extracts and 60 min for the olive leaves extract.

## 4. Conclusions

Different extraction methods were applied to identify the best solvent mixture to extract secoiridoids, where the MeOH/H_2_O 80/20 mixture was found to be the best one. *q*NMR was applied for the quantification of oleocanthal in extra virgin olive oils and the obtained results were in good agreement with the HPLC analysis, demonstrating that this is a simple and reliable method that does not require large quantities of solvent to determine the content of oleocanthal, as well as other related aldehydes. From the HPLC analyses of the main phenols contributing to the health claim, it was established that olive oils from the Bianchera cultivar were rich in phenols and satisfy the health claim. The total phenolic content (TPC) was determined for six olive oils from the Bianchera cultivar and it was demonstrated that they had a higher phenolic content relative to other oils and that the TPC can be used as a first approach to determine whether the olive oil studied can satisfy the health claim. The pomace and olive leaves were also very rich in phenols and can be used as a valuable source of these compounds. In particular, the olive leaves were rich in oleuropein. The antioxidant activity was evaluated using the TEAC/ABTS method and it was shown that extraction of the olive leaves with MeOH/H_2_O 80/20 gave the highest level of phenols content, as well as the highest antioxidant activity.

## Figures and Tables

**Table 1:** ^1^H NMR chemical shift (c.s.) of aldehydic protons.

Compound	c.s. ^a^ (ppm)
4-nitrobenzaldehyde (NB)	10.15
**7**	9.51
**8**	9.52
**9**	9.63, 9.23
**10**	9.64, 9.21

^a^ c.s. chemical shift.

**Table 2:** Quantification of compounds **7**–**10** with different extraction methods by *q*NMR.

Compound	A ^a,b^ (Karkoula) (std) ^g^	B ^c^ (std) ^g^	C ^d^ (std) ^g^	D ^e^ (std) ^g^	E ^f^ (std) ^g^
**7**	78 (10)	73 (9)	564 (51)	48 (6)	351 (38)
**8**	111 (5)	111 (4)	799 (24)	40 (2)	510 (20)
**9**	237 (11)	237 (11)	1324 (53)	69 (3)	884 (35)
**10**	99 (4)	103 (4)	506 (21)	19 (1)	590 (12)
Total	525 (30)	524 (27)	3193 (149)	176 (12)	2335 (105)

All values are expressed as milligrams per kilogram of olive oil (mg/kg). ^a^ Mean value of three different extractions; ^b^ 5 g of olive oil diluted in 20 mL of hexane and 20 mL of acetonitrile; ^c^ 5 g of olive oil diluted in 10 mL of hexane and 10 mL of acetonitrile; ^d^ 5 g of olive oil diluted in 10 mL of hexane and 10 mL of methanol-water 80:20; ^e^ 5 g of olive oil diluted in 10 mL of hexane and 10 mL of methanol-water 50:50; ^f^ 5 g of olive oil diluted in 10 mL of methanol-water 80:20; ^g^ standard deviation.

**Table 3:** Olive oil samples.

Olive Oil Sample	Variety
1	100% Bianchera
2	100% Bianchera
3	100% Bianchera
4	100% Bianchera
5	100% Bianchera
6	100% Bianchera
7	40% Frantoio, 30% Leccino, 10% Pendolino, 5% Miracolo, 5% Itrana, 5% Storta, 5% Carolea
8	50% Frantoio, 45% Leccino, 5% Itrana
9	Commercial olive oil from Puglia Region (blend of unknown cultivars)
10	Commercial olive oil from Calabria Region (blend of unknown cultivars)
11	Commercial olive oil from Tuscany Region (blend of unknown cultivars)
12	Commercial olive oil from EU (blend of unknown cultivars)

**Table 4:** Concentrations of compounds **7**–**10** in milligrams per kilogram of olive oil (mg/kg), as determined using *q*NMR.

Compound	c.s. δ (ppm)	Sample 2	Sample 6	Sample 12
		(mg/kg)	(mg/kg)	(mg/kg)
Oleacein **10**	9.65	354	335	97
Oleocanthal **9**	9.63	768 (789) ^a^	724 (739) ^a^	283 (259) ^a^
3,4-DHPEA-EA **8**	9.53	168	212	197
*p*-HPEA-EA **7**	9.51	68	140	118
Total amount		1358	1411	695

^a^ Values obtained via HPLC analysis using a calibration curve for oleocanthal.

**Table 5:** Quantitative HPLC analyses of samples 2, 6 and 12.

Compound	R_t_ (min)	Sample 2 ^a^	Sample 6 ^a^	Sample 12 ^a^
		(std) ^b^	(std) ^b^	(std) ^b^
Hydroxytyrosol **2**	6.6	0.03 (0.00)	0.02 (0.00)	0.21 (0.00)
Tyrosol **1**	8.9	0.06 (0.00)	0.03 (0.00)	0.17 (0.00)
oleacein **10** + oleuropein **3**	17.2	1.64 (0.01)	1.87 (0.01)	0.44 (0.00)
oleocanthal **9**	22.3–22.8	3.40 (0.03)	3.67 (0.03)	1.03 (0.00)
3,4-DHPEA-EA **8**	24.5	1.19 (0.00)	1.19 (0.00)	1.32 (0.00)
*p*-HPEA-EA **7**	26.4	0.20 (0.00)	0.15 (0.00)	0.31 (0.00)
Total amount		6.52 (0.05)	6.93 (0.06)	3.49 (0.03)

^a^ mg(TE)/20 g olive oil; ^b^ std: standard deviation.

**Table 6:** Total phenolic content (TPC) of olive oils.

Sample	TPC ^g^ mg(GAE)/mL ^h^ (std)	TPC mg(GAE)/kg (std)	TPC mg(GAE)/20 g
1 (Bianchera)	0.0177 (0.0004)	338 (7)	6.76 (0.15)
2 (Bianchera)	0.0203 (0.0019)	401 (36)	8.02 (0.74)
3 (Bianchera)	0.0257 (0.0005)	508 (10)	10.16 (0.19)
4 (Bianchera)	0.0237 (0.0005)	474 (10)	9.48 (0.20)
5 (Bianchera)	0.0193 (0.0005)	386 (10)	7.72 (0.20)
6 (Bianchera)	0.0180 (0.0008)	359 (16)	7.18 (0.31)
7 (blend) ^a^	0.0127 (0.0005)	253 (10)	5.06 (0.20)
8 (blend) ^b^	0.0110 (0.0001)	219 (2)	4.38 (0.04)
9 (blend) ^c^	0.0137 (0.0009)	273 (41)	5.46 (0.82)
10 (blend) ^d^	0.0053 (0.0005)	106 (10)	2.12 (0.20)
11(blend) ^e^	0.0077 (0.0009)	151 (20)	3.02 (0.33)
12 (blend) ^f^	0.0093 (0.0005)	186 (10)	3.72 (0.20)

^a^ 40% Frantoio, 30% Leccino, 10% Pendolino, 5% Miracolo, 5% Itrana, 5% Storta, 5% Carolea; ^b^ 50% Frantoio, 45% Leccino, 5% Itrana; ^c^ commercial olive oil from the Puglia Region; ^d^ commercial olive oil from the Calabria Region; ^e^ commercial olive oil from the Toscana Region; ^f^ commercial olive oil from EU; ^g^ mean value of triplicate analyses; ^h^ GAE: gallic acid equivalent.

**Table 7:** TPC and antioxidant activity.

Sample	TPC (mg(GAE)/mL)	TEAC ^f^/ABTS
Olive oil 2	0.203	4213
Olive oil 6	0.180	3888
Olive oil 12	0.077	3132
Pomace	0.017 ^a^	1515
	0.008 ^b^	3203
	0.017 ^c^	1741
Olive leaves	0.023 ^a^	1192
	0.019 ^d^	863
	0.022 ^e^	1085

^a^ MeOH/H_2_O 80/20; ^b^ EtOH/H_2_O 80/20; ^c^ EtOH/H_2_O 30/70; ^d^ EtOH/H_2_O 70/30; ^e^ H_2_O 100%; ^f^ TEAC: Trolox equivalent antioxidant capacity.

**Table 8:** Gradient for HPLC analyses.

Time (min)	Solvent A (%)	Solvent B (%)
0	97	3
10	78	23
15	78	23
22	50	50
27	2	98
33	2	98
45	97	3
55	97	3

**Table 9:** Gradient for semi-preparative HPLC analyses.

Time (min)	Solvent A (%)	Solvent B (%)
0	97	3
40	2	98
50	97	3
60	97	3

## Data Availability

The data presented in this study are available within the article and in Supplementary Materials.

## Supplementary Materials

The following are available online, Figure S1: calibration curve of oleocanthal for NMR quantification, Figure S2: calibration curve of tyrosol for HPLC quantification, Figure S3: calibration curve of oleocanthal for HPLC quantification, Figure S4: calibration curve of gallic acid, Figure S5: Determination of the stationary state of ABTS for sample 2, Figure S6: Determination of the stationary state of ABTS for pomace extract, Figure S7: Determination of the stationary state of ABTS for olive leaves extract, Figure S8: calibration curve of Trolox.

## Sample Availability

Samples of the compounds are not available.
