# Supplementary material for: Oleocanthal Quantification Using 1H NMR Spectroscopy and Polyphenols HPLC Analysis of Olive Oil from the Bianchera/Belica Cultivar
Source: Molecules. 2021 Jan 5;26(1):242. doi: 10.3390/molecules26010242 (PMC7796514; doi:10.3390/molecules26010242)
Supplement: Supplementary file 1 [file molecules-26-00242-s001.pdf]

Oleocanthal Quantification Using ^{1}HNMR Spectroscopy and Polyphenols HPLC Analysis of Olive Oil from the Biancheria/Belica Cultivar

Martina Starec, Antonella Calabretti, Federico Berti and Cristina Forzato*

Supplementary materials

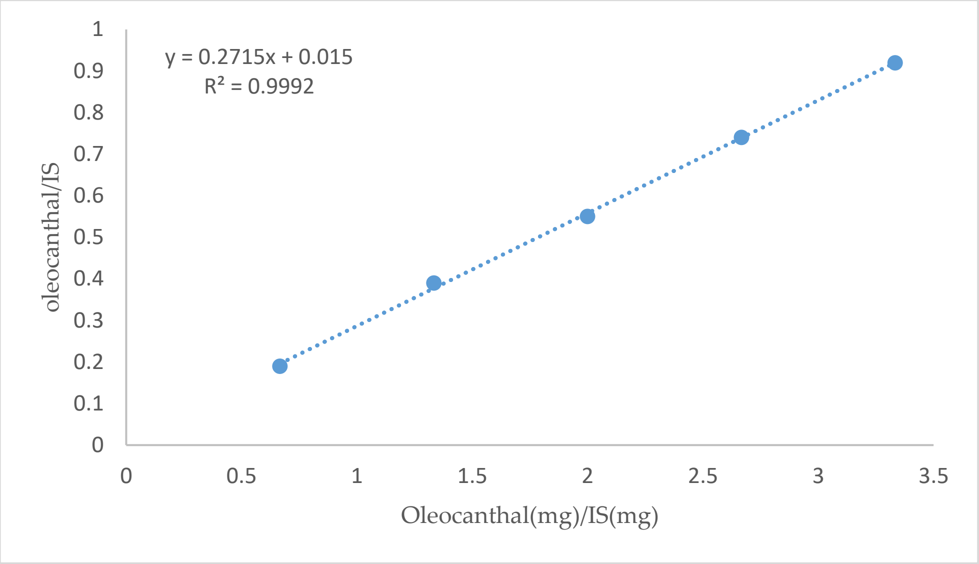

Figure S1 – calibration curve of oleocanthal for NMR quantification

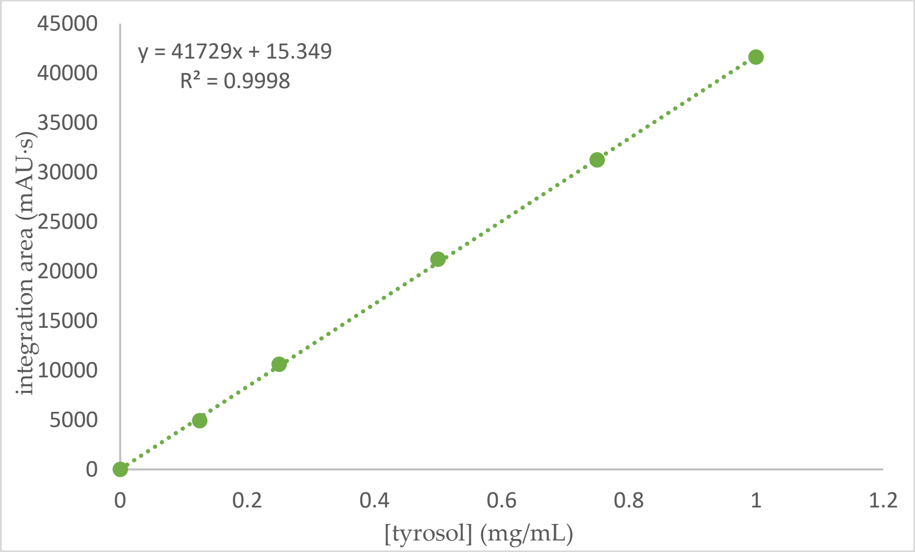

Figure S2 – calibration curve of tyrosol for HPLC quantification

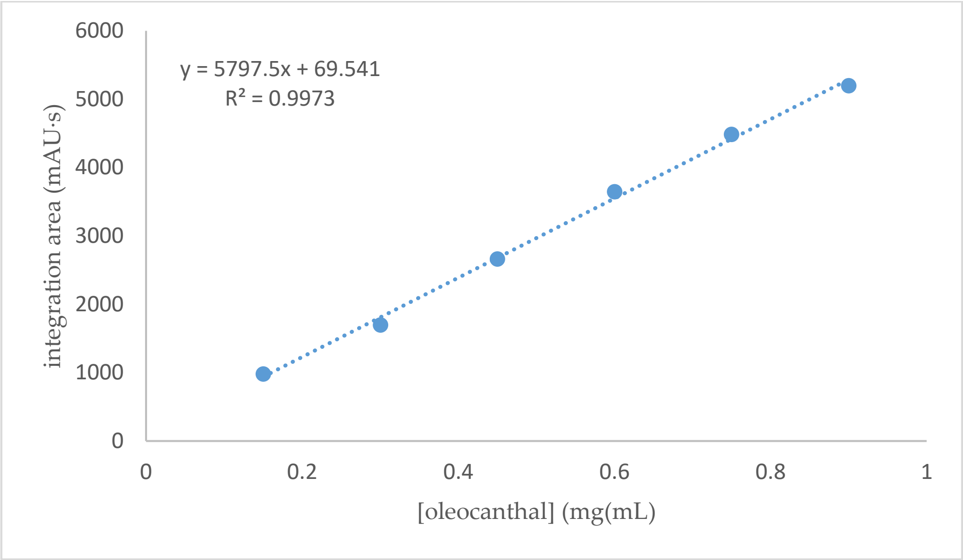

Figure S3 – calibration curve of oleocanthal for HPLC quantification

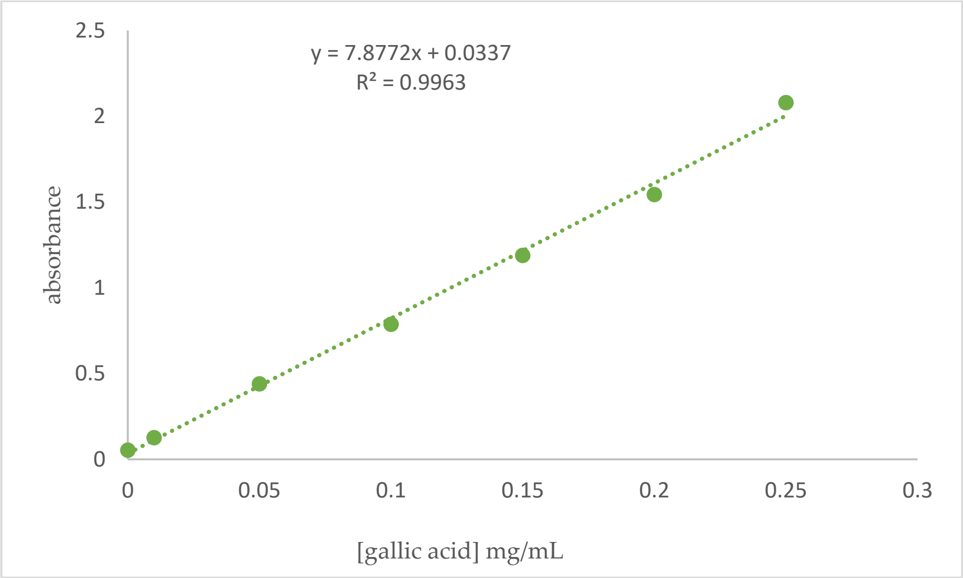

Figure S4 – calibration curve of gallic acid

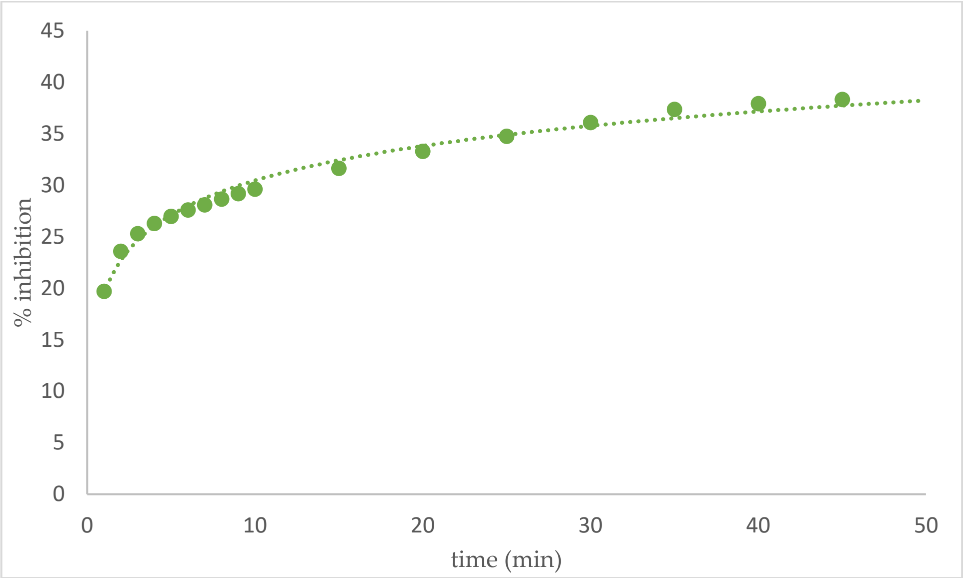

Figure S5 – Determination of the stationary state of ABTS for sample 2

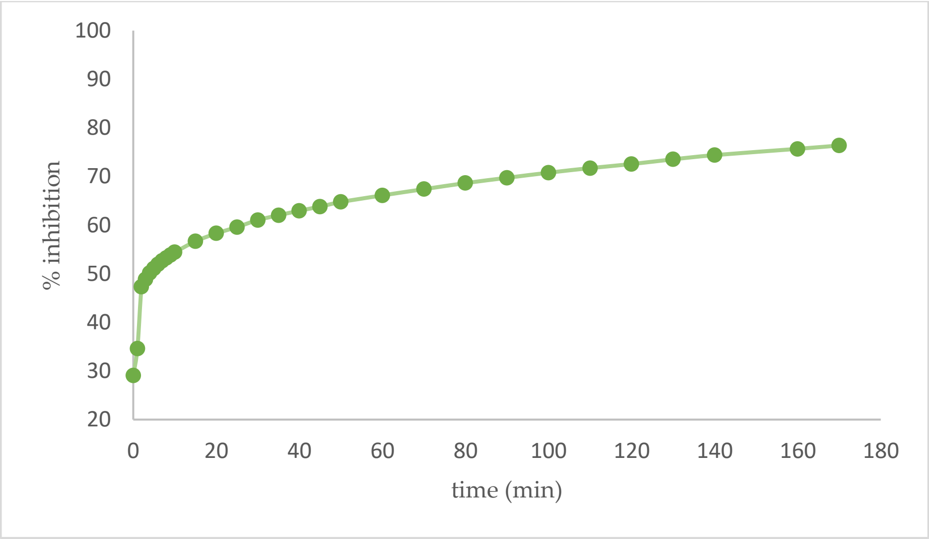

Figure S6 – Determination of the stationary state of ABTS for pomace extract

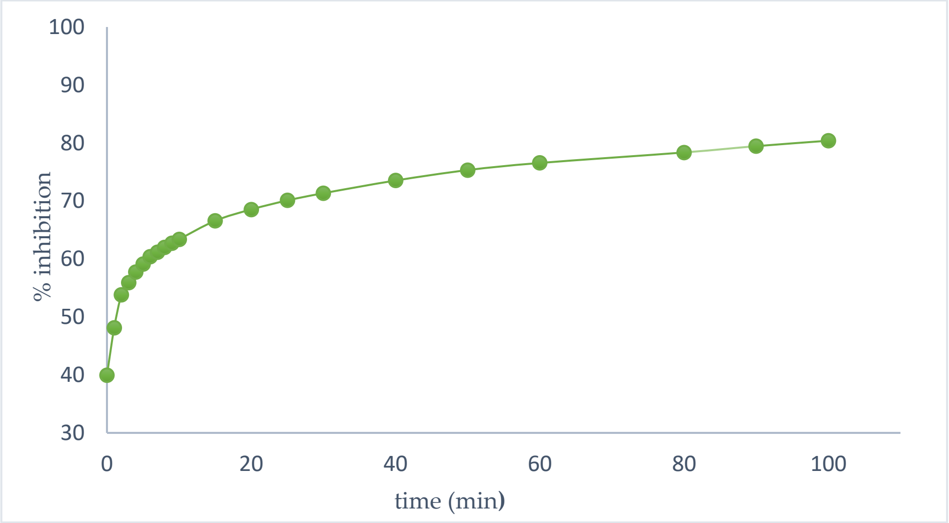

Figure S7 – Determination of the stationary state of ABTS for olive leaves extract

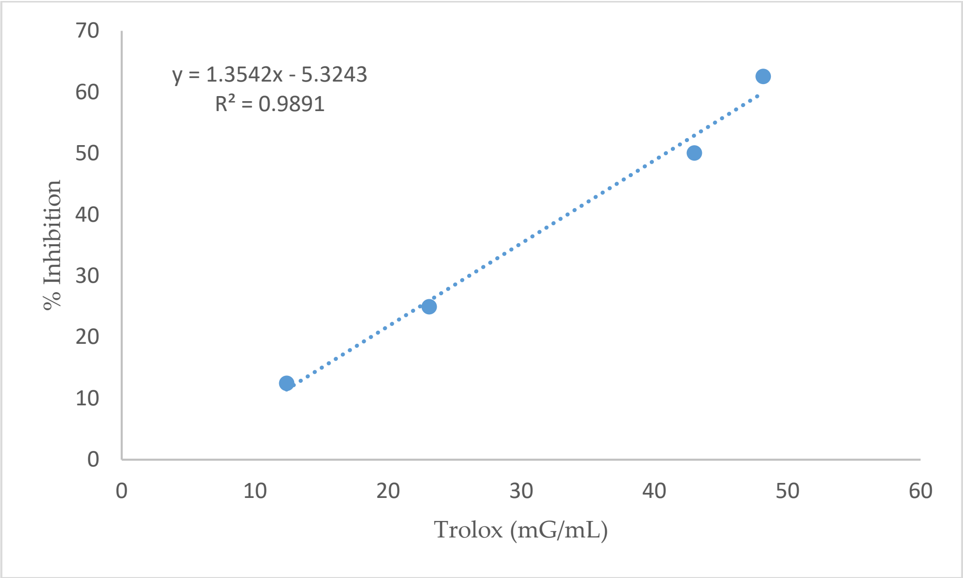

Figure S8 – calibration curve of Trolox
